# Molecular diagnosis of Chagas disease: a systematic review and meta-analysis

**DOI:** 10.1186/s40249-023-01143-7

**Published:** 2023-10-16

**Authors:** Guillermo Pascual-Vázquez, Montserrat Alonso-Sardón, Beatriz Rodríguez-Alonso, Javier Pardo-Lledías, Angela Romero Alegría, Pedro Fernández-Soto, Juan Luis Muñoz Bellido, Antonio Muro, Moncef Belhassen-García

**Affiliations:** 1 Infectious and Tropical Diseases Group (E-INTRO), Biomedical Research Institute of Salamanca (IBSAL), Tropical Diseases Research Center of the University of Salamanca (CIETUS), Faculty of Pharmacy, University of Salamanca, Salamanca, Spain; 2 Area of Preventive Medicine, Epidemiology and Public Health, Department of Biomedical and Diagnostic Sciences, Faculty of Medicine, E-INTRO, IBSAL, CIETUS, University of Salamanca, Salamanca, Spain; 3 Internal Medicine Service, University Health Care Complex of Salamanca (CAUSA), E-INTRO, IBSAL, CIETUS, Salamanca, Spain; 4 Internal Medicine Department, Hospital Marqués de Valdecilla, University of Cantabria, IDIVAL (Valdecilla Research Institute), Santander, Spain; 5 Internal Medicine Service, CAUSA. E-INTRO, IBSAL, CIETUS, Salamanca, Spain; 6 Infectious and Tropical Diseases Group (E-INTRO), IBSAL. CIETUS. Faculty of Pharmacy, University of Salamanca, Salamanca, Spain; 7 Microbiology and Parasitology Service, CAUSA, IBSAL, CIETUS, CSIC, Department of Biomedical and Diagnostic Sciences, University of Salamanca, Salamanca, Spain; 8 Internal Medicine Service. Infectious Diseases Section, CAUSA, IBSAL, CIETUS, University of Salamanca, Paseo San Vicente 58-182, 37007 Salamanca, Spain

**Keywords:** Chagas disease, *Trypanosoma cruzi*, Molecular diagnosis, Polymerase chain reaction, Loop-mediated isothermal amplification

## Abstract

**Background:**

The complexity of the Chagas disease and its phases is impossible to have a unique test for both phases and a lot of different epidemiological scenarios. Currently, serology is the reference standard technique; occasionally, results are inconclusive, and a different diagnostic technique is needed. Some guidelines recommend molecular testing. A systematic review and meta-analysis of available molecular tools/techniques for the diagnosis of Chagas disease was performed to measure their heterogeneity and efficacy in detecting *Trypanosoma cruzi* infection in blood samples.

**Methods:**

A systematic review was conducted up to July 27, 2022, including studies published in international databases. Inclusion and exclusion criteria were defined to select eligible studies. Data were extracted and presented according to PRISMA 2020 guidelines. Study quality was assessed using Quality Assessment of Diagnostic Accuracy Studies-2 (QUADAS-2). A random-effects model was used to calculate pooled sensitivity, specificity, and diagnostic odds ratio (*DOR*). Forest plots and a summary of the receiving operating characteristics (SROC) curves displayed the outcomes. Heterogeneity was determined by* I*^2^ and Tau^2^ statistics and *P* values. Funnel plots and Deek's test were used to assess publication bias. A quantitative meta-analysis of the different outcomes in the two different clinical phases was performed.

**Results:**

We identified 858 records and selected 32 papers. Studies pertained to endemic countries and nonendemic areas with adult and paediatric populations. The sample sizes ranged from 17 to 708 patients. There were no concerns regarding the risk of bias and applicability of all included studies. A positive and nonsignificant correlation coefficient (*S* = 0.020; *P* = 0.992) was obtained in the set of studies that evaluated diagnostic tests in the acute phase population (ACD). A positive and significant correlation coefficient (*S* = 0.597; *P* < 0.000) was obtained in the case of studies performed in the chronic phase population (CCD). This resulted in high heterogeneity between studies, with the master mix origin and guanidine addition representing significant sources.

**Interpretation/Conclusions and relevance:**

The results described in this meta-analysis (qualitative and quantitative analyses) do not allow the selection of the optimal protocol of molecular method for the study of *Trypanosoma cruzi* infection in any of its phases, among other reasons due to the complexity of this infection. Continuous analysis and optimization of the different molecular techniques is crucial to implement this efficient diagnosis in endemic areas.

**Supplementary Information:**

The online version contains supplementary material available at 10.1186/s40249-023-01143-7.

## Introduction

Chagas disease (CD) is caused by infection with the protozoan parasite *Trypanosoma cruzi* [1, 2], and although most infected patients are asymptomatic, CD is responsible for a higher burden of morbidity and mortality than any other parasitic disease in the Western hemisphere, including malaria. The Pan American Health Organization (PAHO)/World Health Organization (WHO) and other public health authorities consider CD to be a neglected tropical disease (NTD) that mainly affects low-income populations.

Historically, the disease occurred predominantly in rural areas of Latin America, where it is endemic in 21 continental Latin American countries, from the southern United States to the northern regions of Argentina and Chile. Vector-borne transmission occurs exclusively in the Americas, where an estimated 6 million people are infected [3], through repeated exposure of residents of infested houses to infected vectors [2, 4]. Chronic Chagasic cardiomyopathy (CCC) is the most important complication in patients with CD.

CD diagnosis depends on the phase (acute or chronic) in which a patient is found to be infected. Parasitemia is high during the acute phase and the congenital form, as well as in reactivations caused by immunosuppression during the chronic phase. The parasite can be detected by microscopy of peripheral blood by thin or thick smears with Giemsa staining. Sensitivity can be increased by concentration techniques such as microhaematocrit concentration or double centrifugation methods. Techniques such as haemoculture or xenodiagnoses are currently in disuse. Direct parasitological methods usually prove negative in 30–60% of patients in the chronic phase due to minimum parasitemia. For decades, serology has been a useful tool in the diagnosis of the chronic phase in CD, detecting anti-*T. cruzi* IgG antibodies. The WHO defines the diagnosis of the disease during its chronic phase by positivity in two serological tests carried out using different methods serological tests do not offer 100% sensitivity and specificity [5, 6], and none of the techniques described serves as a marker of cure or evolution of the infection, since after treatment, if there is a cure, seroconversion may take many years.

In recent years, PCR (polymerase chain reaction) has been the predominant molecular technique used. PCR has proven useful during acute-phase or chronic-phase reactivations due to its greater sensitivity compared to microscopy methods [7]. The viability of PCR use during the chronic phase is debatable because it yields a positive result in 40–70% of patients who have previously been diagnosed by conventional serological methods, depending on the degree of parasitemia, sample volume, DNA purification, target region, study population characteristics, and great genetic variability between the parasite’s discrete typing units (DTUs). PCR has also been used for follow-up treatment efficacy so that a positive result at the end of treatment would indicate therapeutic failure. Real-time (quantitative) PCR (qPCR) has been developed, enabling parasite DNA detection and quantification from clinical samples, although it exhibits highly variable analytical reliability, specificity, and sensitivity, thereby hampering its standardization for use in routine clinical matters. Such methods that were impracticable in endemic areas with few resources because they are sophisticated techniques, require qualified personnel and are expensive. Currently, after the covid 19 pandemic, the situation has improved, with a greater number of PCR kits being available in laboratories in these areas of the planet, especially in Latin America. It would be advisable to reduce costs to further facilitate the implementation of molecular techniques in low-income countries.

Another technique is loop-mediated isothermal amplification (LAMP), a sensitive, specific molecular method which is simpler, faster, and cheaper than PCR and its variants and must thus be used in CD diagnosis [8]. Such a technique has recently been revealed as an alternative with great potential for diagnosis in endemic areas [9]. The LAMP reaction requires four primers (two inner and two outer primers), which specifically recognize six distinct sequences in target DNA, thus ensuring high specificity for amplification. The amplification process can be divided into two phases. This method operates on the fundamental principle of the production of a large quantity of DNA amplification products with a mutually complementary sequence and an alternating, repeated structure [10]. Progress regarding new LAMP methodologies for CD diagnosis has been described in recent years [9]. However, such laboratory tools are still being developed, and larger amounts of reagents and materials are needed, which could increase the value of diagnosis in communities living in endemic areas. However, the role of LAMP in the diagnosis of CD remains to be clarified.

There is no consensus regarding the most effective molecular protocol for the diagnosis of CD. From a clinical perspective, the complexity of the Chagas disease and its phases is impossible to have a unique test for both phases and a lot of different epidemiological scenarios [11, 12]. Despite the availability of so many tests, there is no consensus on establishing reference techniques, and no single test is considered the gold standard for confirming the diagnosis of infection by this parasite due to complexity of Chagas disease. The diagnostic and therapeutic difficulties presented by this systemic parasitosis require us to continue to strengthen the implementation of strategies and recommendations on the management of trypanosomiasis, such as the updating of clinical guidelines [13]. This study aims to measure the heterogeneity and efficacy of molecular tools/techniques to detect *Trypanosoma cruzi* infection in blood samples.

## Methods

### Research question

This systematic review and meta-analysis were conducted based on *Preferred Reporting Items for Systematic reviews and Meta-Analyses* (PRISMA 2020) Statement (http://www.prisma-statement.org/) [14, 15] recommendations to answer the PICO question: “In patients at risk of *T. cruzi* infection (**P**opulation), which molecular diagnostic method (**I**ntervention) is the most effective for the diagnosis of Chagas disease (**O**utcome) compared to other reference methods (**C**omparison)?”.

### Eligibility criteria

Studies eligible for the analysis were defined using the PICO framework. Articles were selected if they followed the WHO/PAHO recommendations: an individual is diagnosed as infected with *T. cruzi* in the chronic phase of the disease when the results of two serological tests are positive and direct parasitological tests in acute phase or chronic phase confirm reactivation. (i) Diagnosis for patients with suspected chronic *T. cruzi* infection: combining two serological tests with antigens that detect different antibodies against *T. cruzi* plus a third test if there are conflicting results; (ii) diagnosis for seroepidemiological survey to identify patients with chronic Chagas disease: use of the ELISA or immunochromatography test (ICT test); (iii) diagnosis for patients with suspected acute *T. cruzi* infection: direct parasitological tests (direct observation) and subsequent serological follow-up; (iv) diagnosis in haemotherapy services: ELISA or chemiluminescent microparticle immunoassay (CMIA) tests [16].

*Inclusion criteria* for the present review consisted of: (i) peer-reviewed articles containing original data; (ii) accessible full text and abstract; (iii) cross-sectional studies reporting the diagnostic efficacy of different molecular techniques in clinical blood patient samples; (iv) research had to examine patients with Chagas infection vs. noninfected patients; (v) studies in patients in acute phase, chronic phase or congenital transmission; and (vi) papers had to collect enough quantitative results to extract or calculate false positives, false negatives, true positives and true negatives. Literature that did not satisfy these criteria was excluded.

*Exclusion criteria* were as follows: (i) research on nonhuman blood samples; (ii) validation therapy studies; (iii) research in immunocompromised populations; (iv) case series without original data; (v) articles with ambiguous/undetermined conclusions; (vi) duplicate publications were removed; and (vii) review articles, opinion articles, letters, case reports, conferences, randomized controlled trials and workshop reports were excluded.

### Information sources and search strategy

MEDLINE/PubMed, Web of Science, EMBASE, SCOPUS and LILACS databases were searched from inception to July 27, 2022. In each electronic database, various combinations of the following search terms were used: (i) “Chagas disease” [MeSH terms] OR “*Trypanosoma cruzi*” [MeSH terms] AND “Molecular diagnosis” [All Fields] AND “PCR” [All Fields] OR “Polymerase chain reaction” [MeSH terms]; (ii) “Chagas disease” [MeSH terms] OR “*Trypanosoma cruzi*” [MeSH terms] AND “Molecular diagnosis” [All Fields] AND “LAMP” [All Fields] OR “Loop-mediated Isothermal Amplification” [MeSH terms]. The following filters were used: “Humans”, “Journal Articles”, and “English OR Spanish language”. We also used references of included primary articles for the search. Searching and collecting the relevant papers were performed by two authors (GPV and MAS). Disagreements between the reviewers were resolved with discussions between the two authors in a joint session, and if an agreement was not reached, a decision was made by a third author (MBG). At the end of the search, the collected articles were managed with Papers v4.31.1997 ♥ 2011–2022, Digital Science & Research Solutions Inc., 625 Massachusetts Avenue; Cambridge, USA.

### Data collection process and data items

Two independent researchers (GPV and MAS) involved in the search conducted an initial screening of the primary citations obtained from databases by title and abstract. After removing duplicates and irrelevant records, following the eligibility and inclusion criteria, the eligible records were selected for full-text download. Using a Microsoft Excel® spreadsheet, these two authors extracted the requisite data: first author name, year of publication, country where the study was conducted, location (endemic or nonendemic area), type of study, study population (children, adults, immunosuppressed), sensitivity, specificity, diagnostic method(s) used, molecular target, genetic material extraction technique, type of sample, and data on amplification and development processes. Disagreement was resolved by discussion with a third author (MBG).

### Study risk of bias assessment

Methodological assessment of each study included in the quantitative synthesis was conducted using the Quality Assessment of Diagnostic Accuracy Studies (QUADAS-2) tool [17] by two independent reviewers (GPV and MAS). This tool comprises 4 domains: patient selection; index test; reference standard; and flow of participants through the study and timing of tests. Each domain is assessed in terms of risk of bias, and the first 3 domains are also assessed in terms of concerns regarding applicability. Conflicts were resolved by discussion and involvement of a third senior author (MBG).

### Data synthesis and statistical analyses

For each article included in this review, we extracted the first author, publication year, design type, region, population, sample size, laboratory technique, sensitivity, specificity, type of sample, genetic material extraction technique and molecular target. A table was compiled with pooled data on the main study characteristics. Statistical analysis was conducted according to the following process: 1st, statistical pooling/clustering of diagnostic efficacy variables; 2nd, heterogeneity study among/between bibliographic records; 3rd, explaining the heterogeneity source among/between included studies; and 4th, graphical display/presentation of results. DerSimonian and Laird random-effects models with inverse-variance weights, taking heterogeneity and threshold effect into account (defined as the possibility of subjective bias in declaring a result as positive), were applied to analyse pooled data and plot the diagnostic performance measurements across studies: sensitivity, specificity, positive likelihood ratio (+*LR*), negative likelihood ratio (*−LR*) and diagnostic odds ratio (*DOR*). Thus, to analyse the threshold effect, the relationship between the sensitivity and specificity of the studies was studied by calculating Spearman's correlation coefficient; if a threshold effect exists, there will be an inverse correlation between the two variables, which is stronger when the threshold effect is greater. Hierarchical summary receiver operating characteristic (*HSROC*) curves were created and applied to visually display the pooled sensitivity, specificity and their 95% confidence intervals (*CIs*). Cochran’s *Q* statistic and Higgins *I*^2^ statistic were used to assess the magnitude of heterogeneity among the included studies (with *I*^2^ values of < 25%, 25–50% and > 50% considered low, moderate and high heterogeneity, respectively). The *I*^2^ was estimated for sensitivity, specificity, and *DOR*; statistically significant heterogeneity was considered for *P* < 0.05 and *I*^2^ > 50%. All statistical analyses were performed with Meta-DiSc 2.0, a web application for meta-analysis of diagnostic test accuracy data.

## Results

### Study selection

The first search identified a total of 858 records (363 in MEDLINE/PubMed, 187 in Web of Science, 175 in EMBASE, 117 in SCOPUS, and 16 in LILACS); 611 studies were removed before screening, and 247 papers remained. In total, 71 articles were not considered relevant after title and abstract screening (52) or were not retrieved (19); 176 studies that met the inclusion criteria were reviewed in depth, and 147 studies were excluded for reasons such as lack of diagnostic accuracy data (69), samples other than blood samples (56), treatment monitoring (13), and immunocompromised patients (9). Three records identified by citation searching were added. Finally, 32 papers were found to be eligible for review and meta-analysis (see PRISMA 2020 flow diagram, Additional file 3: Fig. S1). Bibliographic references were ordered alphabetically; in the case of more than one reference by the same author, they were ordered by year of publication in ascending order [5–9, 11, 18–43].

### Study characteristics

**Fig. 1:**
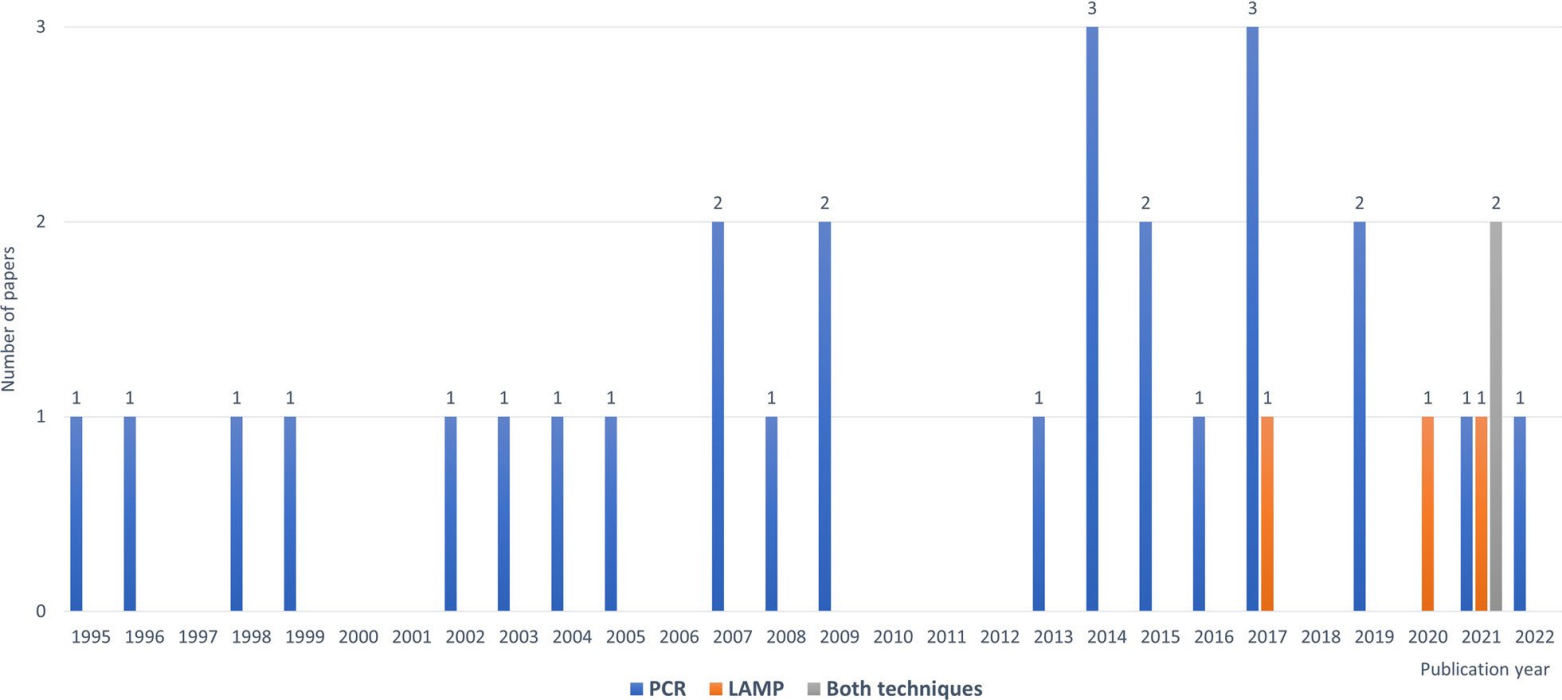
Graph of the number of papers included in this systematic review

Of the 32 study papers, 27 papers assessed the diagnostic performance of PCR techniques [5–7, 11, 18, 21–42], 3 articles assessed *T. cruzi-*LAMP technology [8, 9, 19], and two studies evaluated both molecular techniques [20, 43]. The characteristics of the included studies are outlined in Additional file 1: Table S1 (PCR) and Additional file 2: Table S2 (LAMP). They were published between 1995 [21] and 2022 [42]. An active interest in this research topic has continued in recent years. Based on the year of publication, the bar chart in Fig. 1 illustrates this increasing trend in journal articles. Regarding study design of the studies included in this review, they are longitudinal comparative studies (some of them multicentre, e.g., Benatar et al. [18], or international, e.g., Ramírez et al*.* [11]), some with prospective follow-up, e.g., [6, 18, 20, 24, 34, 35, 38, 41, 43], and others retrospective (case‒control), e.g., [8, 9, 19, 22, 23, 26, 28, 33, 37]. With respect to Hernández et al. [31], the sample collection was both retrospective (for the period 2004–2011) and prospective (for the period 2012–2015). Some studies (the older ones, e.g., [5, 7, 21, 27]) do not clearly define the study design.

### Publication bias/Risk of bias in studies

**Fig. 2:**
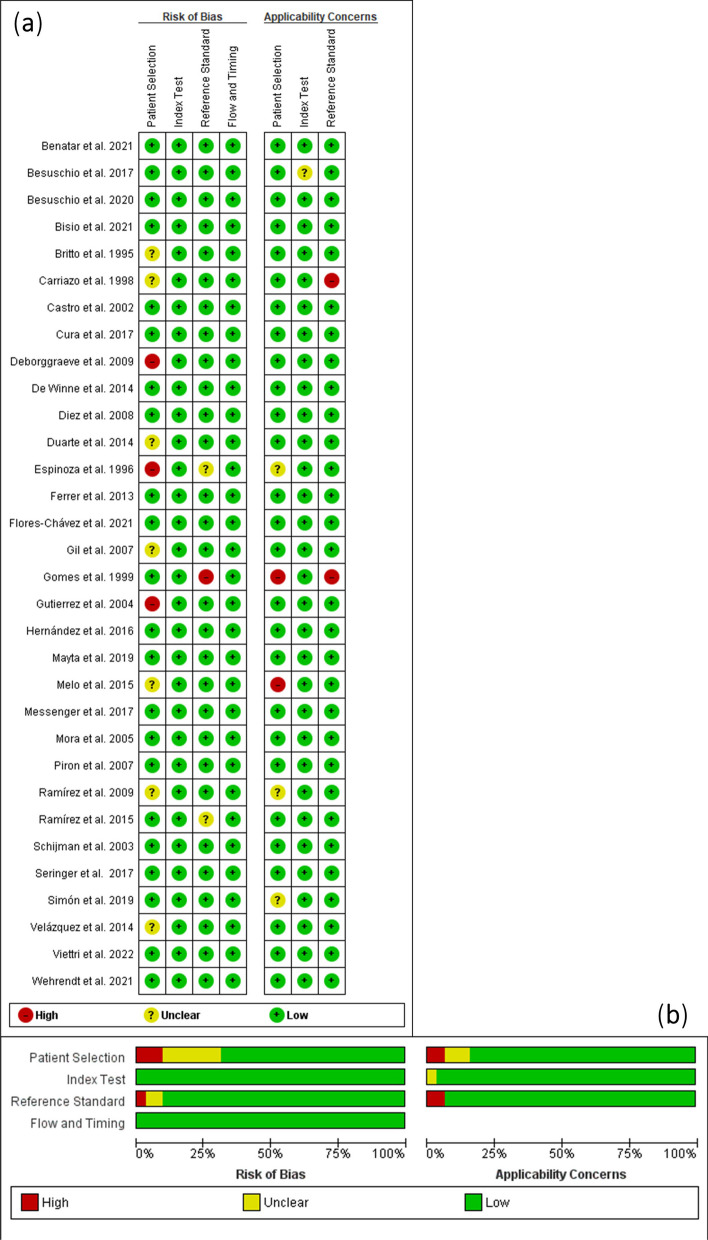
Study quality assessment using the QUADAS-2 scale: **a** Quality evaluation of the individual included studies. **b** Risk of bias and applicability concerns graphical summary

A summary of the study quality assessment using the QUADAS-2 scale can be found in Fig. 2, which shows the quality evaluation of the individual included studies (Fig. 2a) and the risk of bias and applicability concerns of the included studies (Fig. 2b). The risk of bias assessment revealed that most studies carried a low risk of bias. In addition, applicability concerns were also low. Regarding the patient selection aspects, 22 out of 32 studies had a low risk of bias, 7 studies were judged to be unclear, and 3 had a high risk of bias. This result can be explained by the fact that these studies were the oldest and reported insufficient endpoints. In the index test assessment, all studies had a low risk of bias because they clearly mentioned the extraction of data for the index test. Regarding reference standard tests, 29 out of 32 studies were determined to have a low risk of bias, and 30 out of 32 were of low concern in terms of their applicability. For the flow and timing aspects, all studies demonstrated/yielded a low risk of bias in statements regarding the interval time between the reference test and the index test. Therefore, we considered the overall risk of bias to be low, and all included studies generated only low concern regarding applicability in all aspects. This risk of bias disappeared when we analysed subgroups according to diagnostic technique. Thus, the risk of bias only appeared in the cPCR and chronic phase studies.

### Qualitative synthesis: results of individual studies

Detailed information on the 32 included studies is summarized in Additional file 1: Table S1 and Additional file 2: Table S2. They were carried out both in endemic areas (Bolivia, Argentina, Brazil, Chile, Colombia, Venezuela) and in nonendemic areas (Spain). The studies carried out in nonendemic areas always included an immigrant population in the sample. All studies indicate the type of population, sample size and the patients' clinical phase, but in many of them, information on the clinical characteristics and description of the samples is not available. Participants were children/infants/neonates born to seropositive mothers (congenital ACD), e.g., [6, 9, 18, 35, 40, 43], and adult patients in the chronic phase, e.g., [27, 37, 39] (patients with Chagasic cardiomyopathy in Duarte et al. [26]), and/or acute phase, e.g., [28, 31, 36, 42]; controls were included for other diseases (leishmaniasis, malaria, toxoplasmosis, etc.), e.g., [36, 42]), infants born to noninfected mothers and healthy individuals from endemic or nonendemic areas, e.g., [36, 42]. The sample size described in the studies varied from 17 in Diez et al*.* [6] to 708 participants in Hernández et al. [31].

The studies also included information on the type of sample collected from the patients (in all cases, these were blood samples) and the number of samples taken. The blood volume taken ranged from 0.5 mL [20, 23] to 15 mL [7]; 22 of the 32 (68.7%) studies indicated the preservation conditions of the samples; none reported the time period between sample collection and extraction or performance of the diagnostic tests. A total of 6 studies (18.7%) did not specify the type of amplification control of the tests analysed. Only 13 studies (40.6%) indicated the strains used as positive amplification controls; the rest merely indicated the different DTUs used as controls. The parasite load of patients was estimated in 12 of the 32 studies analysed (37.5%). Most of the included studies used proprietary methods except for the studies validating commercial LAMP methods [8, 9, 19, 20, 43].

All studies agree on the limited practical usefulness of molecular diagnostic techniques in patients in the chronic phase (CCD) and confirm their high applicability in acute cases (ACD). The most effective molecular targets are the variable region of kinetoplast DNA (kDNA) and the satellite regions of nuclear DNA. Some individual findings, such as those of the qPCR method, are more reliable than cPCR for CD diagnosis (Cura et al*.* [23]); the commercial qPCR kit is more efficient in congenital Chagas disease diagnosis (Benatar et al. [18]); the kDNA OligoC-TesT showed a significantly higher sensitivity than satDNA OligoC-TesT (De Winner et al. [24]*.* and Ramírez et al*.* [11]); and the Tc24-based PCR assay is more sensitive than that based on kDNA (Espinoza et al*.* [27]) or TcH2AF/R PCR (Gil et al*.* [29]). In addition, LAMP kits offer appropriate analytical sensitivity for the diagnosis of CD patients and are potentially useful for monitoring treatment response (Besuschio et al*.*, 2017 and 2020 [9, 19]); they also offer high performance for the diagnosis of congenital CD (Bisio et al*.* [20], Flores-Chavez et al*.* [8] and Wehrendt et al. [43]).

### Quantitative synthesis: meta-analysis

#### 1st Statistical pooling/clustering of diagnostic efficacy variables

**Fig. 3:**
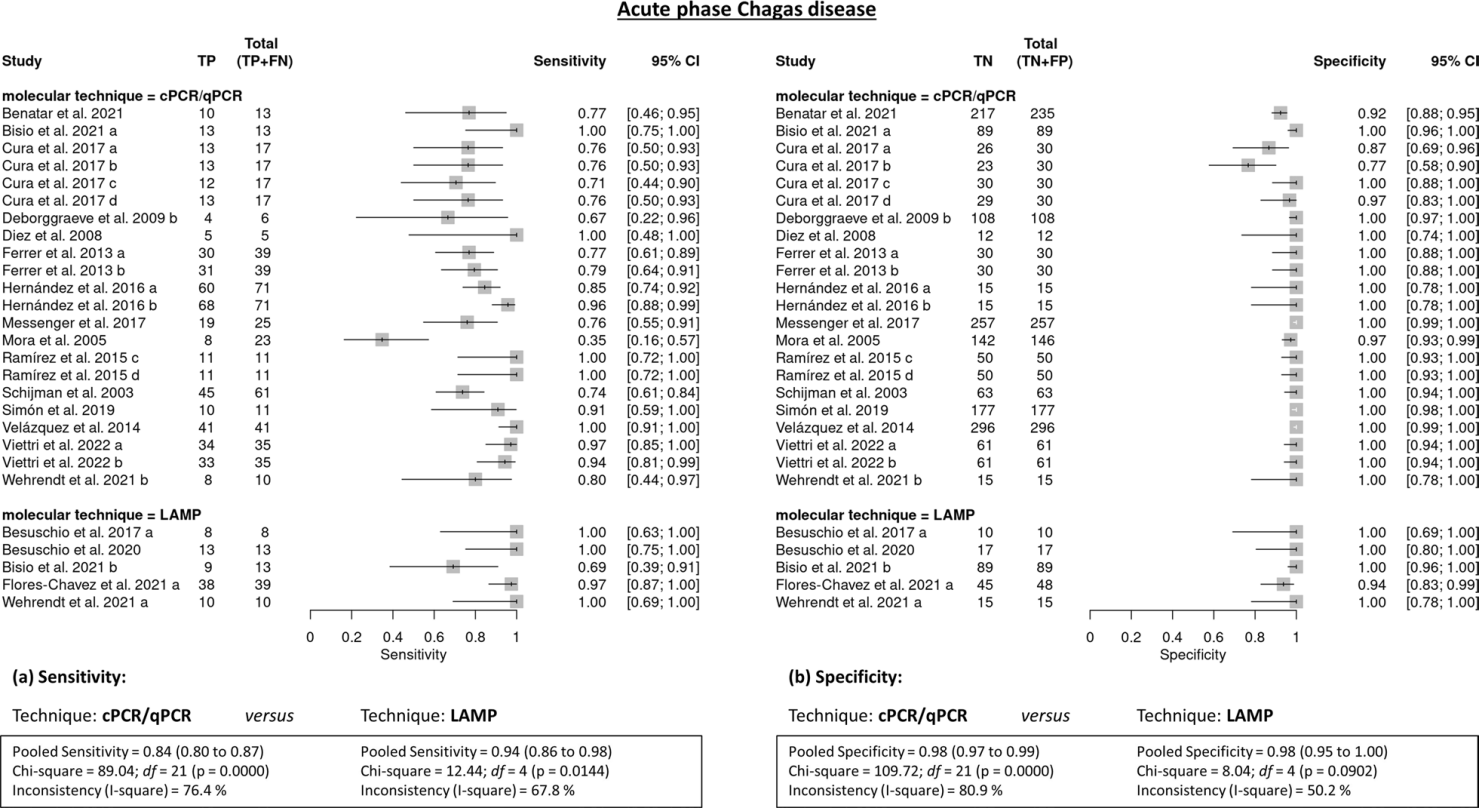
Forest plots with imputed studies testing molecular methods in acute phase Chagas disease (ACD) diagnosis, PCR/qPCR versus LAMP techniques: **a** sensitivity and **b** specificity of the studies

*Acute phase* The set of molecular techniques showed a pooled sensitivity of 84.9% (95% CI 82–87.6) and a pooled specificity of 98.5% (95% CI 97.8–99) in the included studies. Heterogeneity among the studies was very high (*I*^2^ = 76.1% and 77.9%, respectively; *P* < 0.001). The pooled diagnostic odds ratios (*DORs*) of 276.2 (95% CI 122.6–622) for the acute phase are summarized in Table 1. The pooled sensitivity in PCR/qPCR studies was 84% (95% CI 80–87) *versus* 94% (95% CI 86–98) in LAMP studies; the heterogeneity (*I*^2^) among the studies was 76.4% (PCR) and 67.8% (LAMP) (*P* < 0.001 and *P* = 0.014, respectively). Specificity was the same (98%) in the PCR/qPCR and LAMP studies; however, heterogeneity among the PCR/qPCR studies was high (*I*^2^ = 80.9%¸ *P* < 0.001) and lower in the LAMP studies (*I*^2^ = 50.2%¸ *P* = 0.090) (Fig. 3).

**Table 1:** Tabular results: pooled acute phase (ACD)

Acute phase	Summary sensitivity				Summary specificity				Summary DOR (Random effects model)		
Study	Sen	95% CI	TP/(TP + FN)	TN/(TN + FP)	Spe	95% CI	TP/(TP + FN)	TN/(TN + FP)	DOR	95% CI	% Weight
Benatar, 2021	0.769	0.462–0.950	10/13	217/235	0.923	0.882–0.954	10/13	217/235	40.185	10.141–159.230	5.90
Besuschio, 2017 (a)	1.000	0.631–1.000	8/8	10/10	1.000	0.692–1.000	8/8	10/10	357.000	6.391–19,943.000	2.59
Besuschio, 2020	1.000	0.753–1.000	13/13	17/17	1.000	0.805–1.000	13/13	17/17	945.000	17.592–50,761.900	2.62
Bisio, 2021 (a)	1.000	0.753–1.000	13/13	89/89	1.000	0.959–1.000	13/13	89/89	4833.000	91.995–253,904.400	2.64
Bisio, 2021 (b)	0.692	0.386–0.909	9/13	89/89	1.000	0.959–1.000	9/13	89/89	377.890	18.865–7569.800	3.61
Cura, 2017 (a)	0.765	0.501–0.932	13/17	26/30	0.867	0.693–0.962	13/17	26/30	21.125	4.540–98.296	5.66
Cura, 2017 (b)	0.765	0.501–0.932	13/17	23/30	0.767	0.577–0.901	13/17	23/30	10.679	2.622–43.484	5.86
Cura, 2017 (c)	0.706	0.440–0.897	12/17	30/30	1.000	0.884–1.000	12/17	30/30	138.640	7.120–2699.500	3.64
Cura, 2017 (d)	0.765	0.501–0.932	13/17	29/30	0.967	0.828–0.999	13/17	29/30	94.250	9.574–927.810	4.54
Deborggraeve, 2009 (a)	0.667	0.223–0.957	4/6	108/108	1.000	0.966–1.000	4/6	108/108	390.600	16.253–9387.100	3.40
Diez, 2008	1.000	0.478–1.000	5/5	12/12	1.000	0.735–1.000	5/5	12/12	275.000	4.809–15724.400	2.57
Ferrer, 2013 (a)	0.769	0.607–0.889	30/39	30/30	1.000	0.884–1.000	30/39	30/30	195.840	10.908–3516.000	3.74
Ferrer, 2013 (b)	0.795	0.635–0.907	31/39	30/30	1.000	0.884–1.000	31/39	30/30	226.060	12.497–4089.200	3.73
Flores-Chavez, 2021 (a)	0.974	0.865–0.999	38/39	45/48	0.938	0.828–0.987	38/39	45/48	570.000	56.919–5708.100	4.52
Hernández, 2016 (a)	0.845	0.740–0.920	60/71	15/15	1.000	0.782–1.000	60/71	15/15	163.090	9.101–2922.400	3.74
Hernández, 2016 (b)	0.958	0.881–0.991	68/71	15/15	1.000	0.782–1.000	68/71	15/15	606.710	29.785–12,358.500	3.59
Messenger, 2017	0.760	0.549–0.906	19/25	257/257	1.000	0.986–1.000	19/25	257/257	1545.000	83.908–28448.000	3.71
Mora, 2005	0.348	0.164–0.573	8/23	142/146	0.973	0.931–0.992	8/23	142/146	18.933	5.094–70.374	5.99
Ramírez, 2015 (a)	1.000	0.715–1.000	11/11	50/50	1.000	0.929–1.000	11/11	50/50	2323.000	43.759–123,317.900	2.63
Ramírez, 2015 (b)	1.000	0.715–1.000	11/11	50/50	1.000	0.929–1.000	11/11	50/50	2323.000	43.759–123,317.900	2.63
Schijman, 2003	0.738	0.609–0.842	45/61	63/63	1.000	0.943–1.000	45/61	63/63	350.210	20.479–5989.000	3.80
Simón, 2019	0.909	0.587–0.998	10/11	177/177	1.000	0.979–1.000	10/11	177/177	2485.000	95.339–64,771.300	3.31
Velázquez, 2014	1.000	0.914–1.000	41/41	296/296	1.000	0.988–1.000	41/41	296/296	49,219.000	963.580–2514065.000	2.66
Viettri, 2022 (a)	0.971	0.851–0.999	34/35	61/61	1.000	0.941–1.000	34/35	61/61	2829.000	112.170–71348.700	3.35
Viettri, 2022 (b)	0.943	0.808–0.993	33/35	61/61	1.000	0.941–1.000	33/35	61/61	1648.200	76.864–35,342.500	3.53
Wehrendt, 2021 (a)	1.000	0.692–1.000	10/10	15/15	1.000	0.782–1.000	10/10	15/15	651.000	11.954–35,452.900	2.61
Wehrendt, 2021 (b)	0.800	0.444–0.975	8/10	15/15	1.000	0.782–1.000	8/10	15/15	105,400	4.519–2458.400	3.43
Pooled	0.849	0.820–0.876			0.982	0.975–0.987			276.190	122.640–621.990	
No. studies	27				27				27		
Heterogeneity (Chi-squared)	108.81 (*df* = 26)				117.79 (*df* = 26)				63.60 (*df* = 26)		
P-value	0.000				0.000				0.000		
Inconsistency (I-square)	76.1%				77.9%				59.1%		
Estimate of between-study variance (Tau-squared)									2.4158

**Fig. 4:**
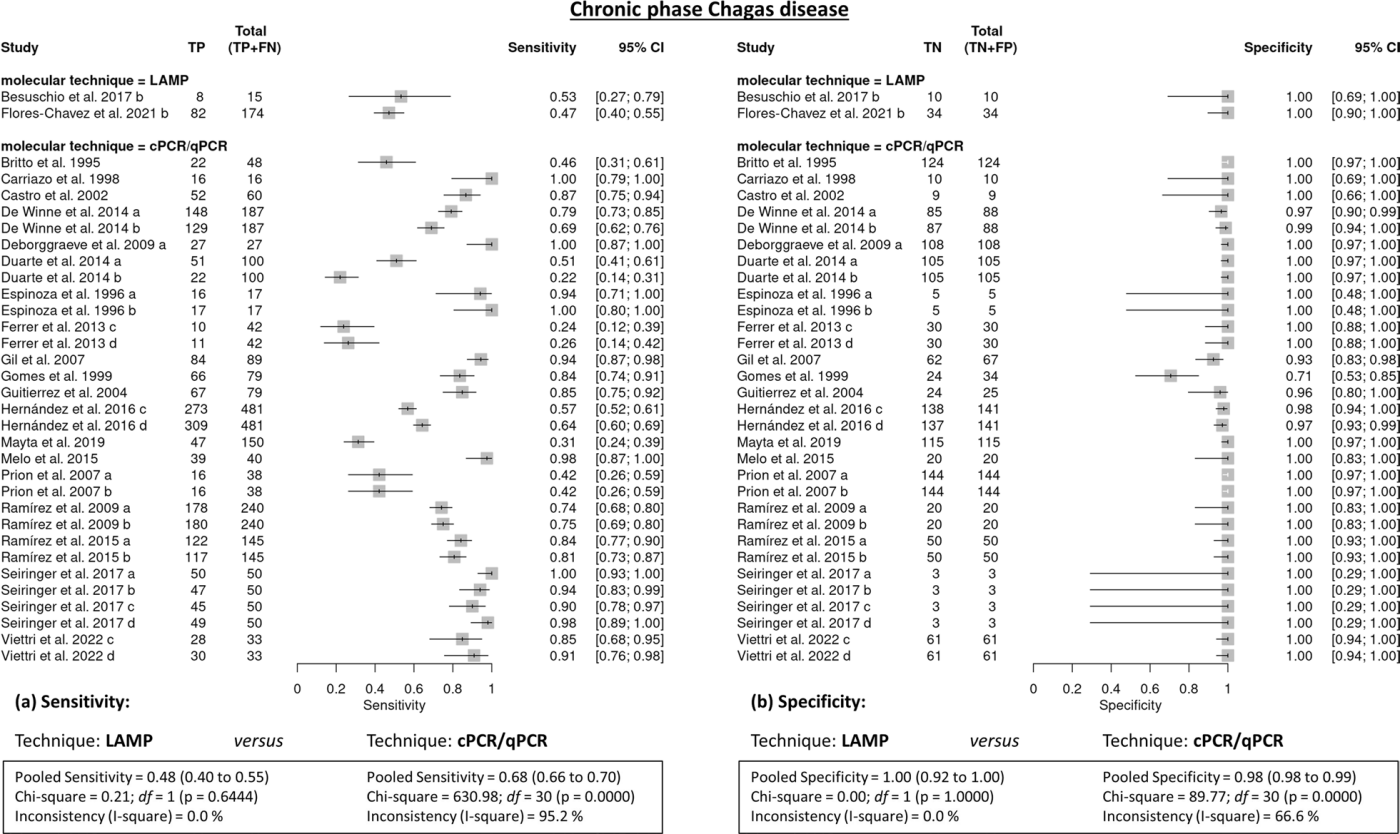
Forest plots with imputed studies testing molecular methods in chronic phase Chagas disease (CCD) diagnosis, PCR/qPCR versus LAMP techniques: **a** sensitivity and **b** specificity of the studies

*Chronic phase* A sensitivity of 67% (95% CI 65.4–68.5) was obtained, and the pooled specificity was very high, at 98.5% (95% CI 97.8–99). The pooled *DOR* was 110.73 (95% CI 69.5–176.5). These results are well below what is desirable for a routine diagnostic method. The heterogeneity of all the diagnostic efficacy variables among the studies with patients in the chronic phase of the disease was found to be very high (*I*^2^ = 95.2% and 64.9%, respectively: *P* < 0.001) (Table 2). In the PCR/qPCR studies, the pooled sensitivity was 68% (95% CI 66–70; I^2^ = 95.2%, *P* < 0.001), and the pooled specificity was 98% (95% CI 98–99; I^2^ = 66.6%, *P* < 0.001); in the LAMP studies, the pooled sensitivity was 48% (95% CI 40–55; *I*^2^ = 0%, *P* = 0.644), and the pooled specificity was 100% (95% CI 92–100;* I*^2^ = 0%, *P* = 1.000) (Fig. 4).

**Table 2:** Tabular results: pooled chronic phase (CCD)

Chronic phase	Summary sensitivity				Summary specificity				Summary DOR (Random effects model)		
Study	Sen	95% CI	TP/(TP + FN)	TN/(TN + FP)	Spe	95% CI	TP/(TP + FN)	TN/(TN + FP)	DOR	95% CI	% Weight
Besuschio, 2017 (b)	0.533	0.266–0.787	8/15	10/10	1.000	0.692–1.000	8/15	10/10	23.800	1.182–479.070	2.06
Britto, 1995	0.447	0.302–0.599	21/47	124/125	0.992	0.956–1.000	21/47	124/125	100.150	12.891–778.110	3.78
Carriazo, 1998	1.000	0.794–1.000	16/16	10/10	1.000	0.692–1.000	16/16	10/10	693.000	12.749–37,669.400	1.24
Castro, 2002	0.867	0.754–0.941	52/60	9/9	1.000	0.664–1.000	52/60	9/9	117.350	6.237–2208.100	2.14
De Winne, 2014 (a)	0.791	0.726–0.847	148/187	85/88	0.966	0.904–0.993	148/187	85/88	107.520	32.248–358.490	7.25
De Winne, 2014 (b)	0.690	0.618–0.755	129/187	87/88	0.989	0.938–1.000	129/187	87/88	193.500	26.308–1423.200	3.93
Deborggraeve, 2009 (b)	1.000	0.872–1.000	27/27	108/108	1.000	0.966–1.000	27/27	108/108	11,935.000	231.600–615039.200	1.27
Duartel, 2014 (a)	0.510	0.408–0.611	51/100	105/105	1.000	0.965–1.000	51/100	105/105	219.530	13.274–3630.400	2.31
Duarte, 2014 (b)	0.220	0.143–0.314	22/100	105/105	1.000	0.965–1.000	22/100	105/105	60.478	3.613–1012.200	2.29
Espinoza, 1996 (a)	0.941	0.713–0.999	16/17	5/5	1.000	0.478–1.000	16/17	5/5	121.000	4.275–3424.600	1.71
Espinoza, 1996 (b)	1.000	0.805–1.000	17/17	5/5	1.000	0.478–1.000	17/17	5/5	385.000	6.807–21776.200	1.22
Ferrer, 2013 (c)	0.238	0.121–0.395	10/42	30/30	1.000	0.884–1.000	10/42	30/30	19.708	1.107–350.990	2.21
Ferrer, 2013 (d)	0.262	0.139–0.420	11/42	30/30	1.000	0.884–1.000	11/42	30/30	22.270	1.257–394.690	2.21
Flores-Chavez, 2021 (b)	0.471	0.395–0.548	82/174	34/34	1.000	0.897–1.000	82/174	34/34	61.541	3.714–1019.700	2.30
Gil, 2007	0.944	0.874–0.982	84/89	62/67	0.925	0.834–0.975	84/89	62/67	208.320	57.788–750.970	6.81
Gomes, 1999	0.835	0.735–0.909	66/79	24/34	0.706	0.525–0.849	66/79	24/34	12.185	4.724–31.430	8.90
Gutierrez, 2004	0.848	0.750–0.919	67/79	24/25	0.960	0.796–0.999	67/79	24/25	134.000	16.531–1086.200	3.67
Hernández, 2016 (c)	0.568	0.522–0.612	273/481	138/141	0.979	0.939–0.996	273/481	138/141	60.375	18.965–192.200	7.53
Hernández, 2016 (d)	0.642	0.598–0.685	309/481	137/141	0.972	0.929–0.992	309/481	137/141	61.531	22.377–169.200	8.46
Mayta, 2019	0.313	0.240–0.394	47/150	115/115	1.000	0.968–1.000	47/150	115/115	106.010	6.453–1741.000	2.32
Melo, 2015	0.975	0.868–0.999	39/40	20/20	1.000	0.832–1.000	39/40	20/20	1079.700	42.082–27700.000	1.80
Piron, 2007 (a)	0.421	0.263–0.592	16/38	144/144	1.000	0.975–1.000	16/38	144/144	211.930	12.279–3657.800	2.25
Piron, 2007 (b)	0.421	0.263–0.592	16/38	144/144	1.000	0.975–1.000	16/38	144/144	211.930	12.279–3657.800	2.25
Ramírez, 2009 (a)	0.742	0.681–0.796	178/240	20/20	1.000	0.832–1.000	178/240	20/20	117.100	6.978–1964.800	2.29
Ramírez, 2009 (b)	0.750	0.690–0.803	180/240	20/20	1.000	0.832–1.000	180/240	20/20	122.320	7.288–2053.200	2.29
Ramírez, 2015 (c)	0,841	0.772–0.897	122/145	50/50	1.000	0.929–1.000	122/145	50/50	526.490	31.374–8835.100	2.29
Ramírez, 2015 (d)	0.807	0.733–0.868	117/145	50/50	1.000	0.929–1.000	117/145	50/50	416.400	24.934–6953.900	2.29
Seiringer, 2017 (a)	1.000	0.929–1.000	50/50	3/3	1.000	0.292–1.000	50/50	3/3	707.000	12.111–41,271.100	1.20
Seiringer, 2017 (b)	0.940	0.835–0.987	47/50	3/3	1.000	0.292–1.000	47/50	3/3	95.000	4.047–2229.800	1.89
Seiringer, 2017 (c)	0.900	0.782–0.967	45/50	3/3	1.000	0.292–1.000	45/50	3/3	57.909	2.628–1275.800	1.96
Seiringer, 2017 (d)	0.980	0.894–0.999	49/50	3/3	1.000	0.292–1.000	49/50	3/3	231.000	7.871–6779.400	1.68
Viettri, 2022 (c)	0.848	0.681–0.949	28/33	61/61	1.000	0.941–1.000	28/33	61/61	637.360	34.070–11923.600	2.15
Viettri, 2022 (d)	0.909	0.757–0.981	30/33	61/61	1.000	0.941–1.000	30/33	61/61	1071.900	53.642–21,417.600	2.07
Pooled	0.670	0.654–0.685			0.985	0.978–0.990			110.730	69.476–176.480	
No. studies	33				33				33		
Heterogeneity (Chi-squared)	663.02 (*df* = 32)				91.13 (*df* = 32)				42.80 (*df* = 32)		
P-value	0.000				0.000				0.096		
Inconsistency (I-square)	95.2%				64.9%				25.2%		
Estimate of between-study variance (Tau-squared)									0.4021

#### 2nd Heterogeneity study

A positive and nonsignificant correlation coefficient (*S* = 0.020, *P* = 0.992) was obtained in the set of studies that evaluated diagnostic tests in the acute phase population (ACD). A positive and significant correlation coefficient (*S* = 0.597, *P* < 0.000) was obtained in the case of studies performed in the chronic phase population (CCD). Therefore, the heterogeneity is not explainable by the threshold effect in studies comprising patients in the acute phase of the disease, and most of the reference tests are qualitative methods in which the results are not very subjective and have little risk of bias. In contrast, there was further indication of a threshold effect in studies of chronic patients, and serological methods are the reference standard techniques for diagnosis, which imposes the requirement of setting threshold values. The forest plots (Figs. 3 and 4) reveal the absence of the bias effect in both clinical phases, and no increase in the sensitivity or specificity of the studies is observed when the other efficacy variable decreases. We ruled out the threshold effect as a source of heterogeneity in the acute phase studies, and it is still recommended that the above explorations for threshold effect are undertaken in chronic phase studies. A threshold effect was detected in subgroups composed of boiled sample and guanidine addition variables.

#### 3rd Analysis of the heterogeneity sources among/between studies

The next step consisted of analysing the relationship between the covariates obtained in the data extraction and the diagnostic efficacy obtained in each study. The existing relationships between sensitivity and false-positive rate (1 − specificity) and the different variables obtained through meta-regressions were analysed.

In the *acute phase,* no statistically significant results were observed in the preparation of the master mixes (*Z* = − 0.561, *P* = 0.575), the molecular technique (*Z* = 1.125, *P* = 0.261), the molecular target (*Z* = − 1.377, *P* = 0.168), guanidine addition (*Z* = − 0.837, *P* = 0.403), or boiling samples (*Z* = − 1.150, *P* = 0.250).

In the *chronic phase,* boiling (*Z* = 2.367, *P* = 0.018) and the addition of guanidine to the blood samples before processing (*Z* = 2.367, *P* = 0.018 and *Z* = 2.256, *P* = 0.020, respectively) were significant sources of heterogeneity. We created subgroups based on the addition of guanidine and boiled samples: “not stored with guanidine and not boiled”, “stored with guanidine and not boiled”, “no guanidine addition data and not boiled” and “no guanidine addition data and boiled”. This new covariate was highly significant (*Z* = 3.250, *P* = 0.001 and *Z* = 2.504, *P* = 0.012).

#### 4th Graphical display/presentation of results

**Fig. 5:**
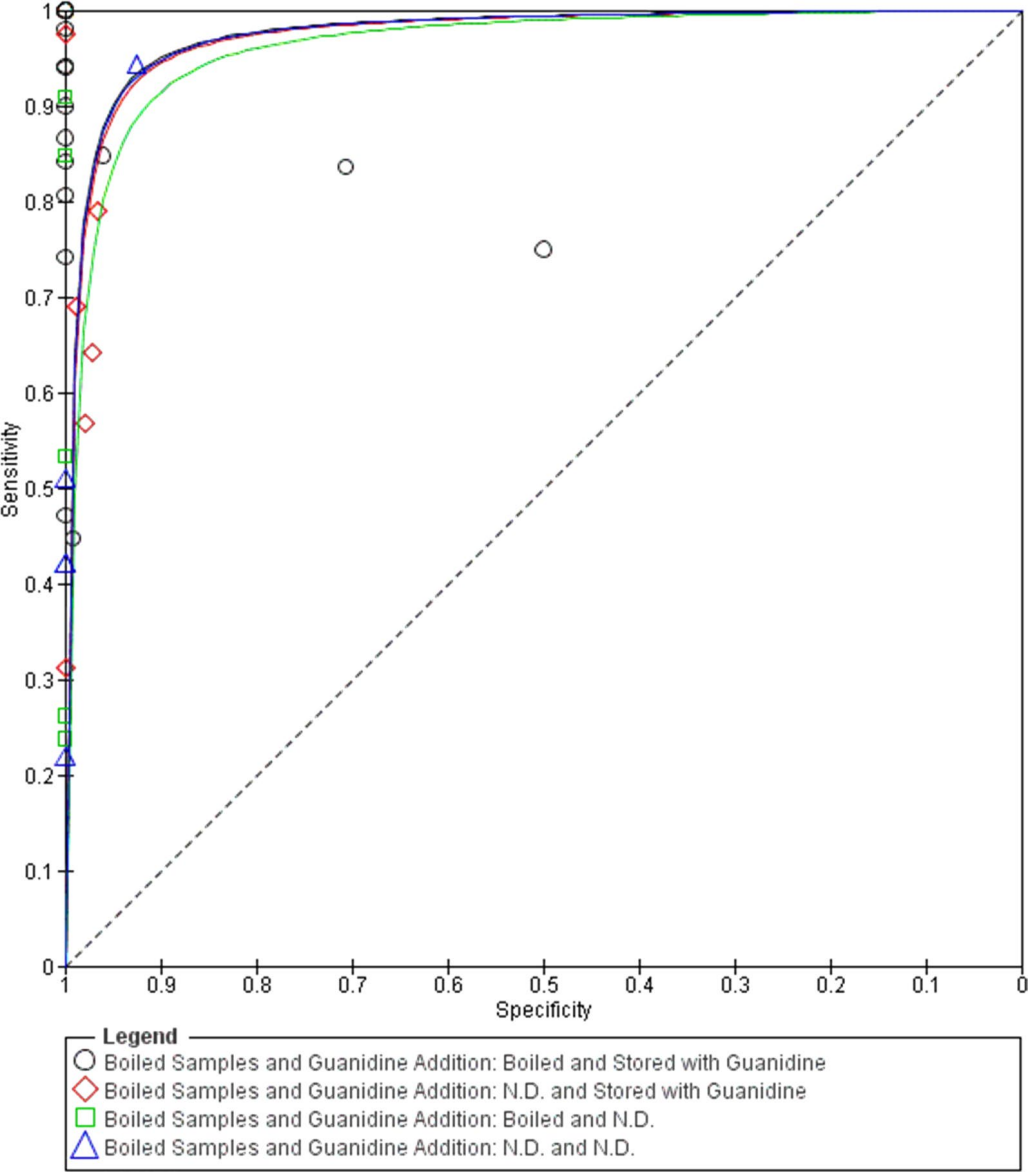
*HSROC* curve of the diagnostic molecular tools: Chronic phase (CCD) patients with subgroups by boiling and addition of guanidine to the blood samples

*HSROC* curves were generated in the two clinical phases grouped according to the variables that showed a significant relationship in the meta-regression analysis. Significant differences in diagnostic efficacy variables were observed only in the *chronic phase* when the studies were divided/grouped according to boiling and addition of guanidine to the sample. Thus, the relationship between sensitivity and *DOR* was positive, i.e., boiling and the addition of guanidine hydrochloride to the sample increased the diagnostic efficacy. The visual representation of the significant results is shown in Fig. 5.

## Discussion

This review and meta-analysis arise from the need to assess the optimal diagnosis of Chagas disease, especially during the chronic phase. Compared to direct diagnostic methods that depend on the observation and identification capacity of health care professionals, PCR has proven to be more sensitive and specific [7]. All the studies analysed confirm the low diagnostic efficacy of molecular techniques in chronic patients. However, these techniques constitute a basic tool in the acute phase. Above all, in cases of congenital transmission, PCR is considered as the reference diagnostic tool. In addition, PCR is essential in detecting reactivations in cases of immunosuppression, transplantation, and transfusion.

Most of the articles reviewed explore the chronic phase diagnosis, while those on acute phase focus on congenital transmission. The articles that analyse the chronic phase assess the possible detection of the parasite in the phase of the disease in which parasitemia is minimal, when direct conventional methods are ineffective. The diagnostic possibilities of molecular methods in the acute phase are focused on nonendemic countries, especially on vertical transmission [44]. *T. cruzi* DNA detection may not be useful for diagnosis in congenital infection when parasite transmission occurs in the last trimester of pregnancy or during delivery due to contact with maternal blood or other fluids from parasitemic mother, therefore during the first month of life of the newborn, the parasitemia could be extremely low or non-existent [45]. In neonates, the presence of the parasite is necessary to obtain an accurate diagnosis. A serological test is decisive, when it is carried out after 9 to 12 months of age, when it has been concluded that maternal *T. cruzi* antibodies have disappeared. Early diagnosis in the acute phase is extremely important due to the high efficacy of treatment of close to 90% [46].

The quality of the studies was analysed using the QUADAS method. Most of the articles included are based on trials in the second phase of validation by calculating the predictive values in a comparative cross-sectional study in subjects with suspicion of the disease or examining routine laboratory methods; therefore, they present some bias in the choice of patients. In many of them, the availability of the reference test result information was not detailed before the index test was performed, nor were they clear in relation to the reference tests, especially in the case of chronic infection. All studies used reference serological methods, although it is necessary to perform other tests to differentiate between acute phase or chronic phase. It is worth noting the lack of information in most of the articles about commercial methods, especially in the sampling and reference tests (Tables 1, 2 and Fig. 2).

The 32 selected studies exhibit high variability of results. This uncertainty depends mainly on infection phase in addition to the different molecular targets, primers, probes, extraction methods and amplification methods, making it difficult to standardize molecular techniques for the diagnosis of CD. In addition, most studies indicate the propensity for errors when *T. rangeli* and *T.* *cruzi* are co-circulating in a same area. Analytical sensitivity is more consistent for kDNA-based PCR *versus* satDNA, depending on the doses present in the different genomes of DTUs [47]. The main problem presented by kDNA as a diagnostic target lies in the enormous number of false positives found in patients infected with *T. rangeli*, since the kinetoplast minicircles seem to be quite conserved within the genus; therefore, in areas where *T. cruzi* and *T. rangeli* are endemic, the use of satDNA PCR is recommended, as *T. rangeli* has few copies of the satellite sequence [48].

Observationally, it can be determined that kDNA-focused molecular diagnostic methods are more sensitive and those based on satDNA are more specific. Multiple comparative studies have been carried out between the different PCR methods for the diagnosis of CD [26, 49]. The international study developed by PAHO and WHO in 2011 stands out [12], in which the diagnostic techniques used in 26 laboratories in 16 countries with dilutions of isolated strains, clinical samples and artificially infected samples are evaluated, defining satDNA and kDNA as the most effective targets. Many studies present inconclusive results that must be analyzed on multiple occasions to determine the infection status in acute infection or to determine the parasitaemia in chronic infection [27, 31, 41]. When the disease becomes chronic, time is not an entirely determining factor, but time could be vital in cases of congenital infections, oral infections, conditions of immunosuppression or for patients undergoing a transplant process.

After the statistical analysis of the diagnostic efficacy of the protocols that apply molecular techniques, it is concluded that they are recommendable for routine diagnosis in the acute phase. These are sufficiently specific and sensitive methods for application (84.9% cumulative sensitivity and 98.5% cumulative specificity). None of the different molecular techniques (cPCR, qPCR and LAMP) present a *DOR* lower than 10, indicating a very high capacity to discriminate between infected and healthy patients [50]. These results are explained by the close relationship between parasitemia and diagnostic efficacy for any direct diagnostic method. In cases of acute infection, parasitemia is relatively high, which makes it possible to detect the pathogen's DNA in most blood samples from patients.

The diagnostic effectiveness of molecular techniques in the chronic phase presents a low sensitivity (cumulative sensitivity of 67%, 95% CI 65.4–68.5) for use as a routine diagnostic tool. This depends on the number of circulating parasites. Furthermore, in the chronic phase, parasitemia is low and intermittent. Therefore, in the processing of the sample from collection to the development of the test, there may be insufficiently high concentrations of parasitic DNA to be amplified. A possible solution to this limitation is the collection of several serial samples from the same patient at different times or an increase in the volume of blood drawn.

In general, the major problem with direct diagnostic techniques, whether molecular or conventional, is their limited application in endemic areas. These are regions in which the vast majority of those infected are in the indeterminate chronic phase of the disease; in these cases, the PCR result is positive for between 50 and 90% of those infected [51].

In addition, the results present great variability depending on multiple factors, such as the patient's parasitaemia, the volume and processing of the sample, the target of the technique or the characteristics of the population [51].

On the other hand, a negative result does not exclude infection, and a serological test would be unavoidable. The entire international community agrees that the most recommendable approach is the combination of both types of diagnosis [28].

The heterogeneity between studies was very high, both in the acute phase (acute: *I*^2^ sensitivity = 76.1% and *I*^2^ specificity = 77.9%, *P* < 0.001) and chronic phase (chronic: *I*^2^ sensitivity = 95.2% and *I*^2^ specificity = 64.9%, *P* < 0.001). This heterogeneity could be explained by different factors that imply a certain patient selection bias and the scarcity of comparative studies. Another determining factor that can explain these contrasts would be the different DTUs circulating in each geographical region in which the studies were carried out, as well as methodological errors in some protocols lacking amplification controls.

The analysis of the heterogeneity of the studies in the chronic phase showed significant relationships between two fundamental variables for the diagnosis of CD: use of the boiling bath and the addition of guanidine to the blood sample. Thus, the addition of guanidine buffer to the blood sample after its collection and the bath in boiling water significantly increased the sensitivity of the technique. This result was confirmed by other meta-analyses carried out on molecular diagnostic techniques for CD, such as that of Brasil et al. [51].

This difference is based on the release of parasite DNA, both by guanidine and by the boiling bath. The addition of guanidine hydrochloride to the sample with EDTA, forming the so-called guanidine-EDTA-blood (GEB), homogenizes the parasite's DNA, inhibits DNases and facilitates sample preservation, even at room temperature. With this procedure, it is possible to detect 1 parasite in up to 10 mL of blood [52].

The boiling water bath has previously been described as an efficient physical method of separation of the DNA networks present in the kinetoplast of the parasite, increasing the homogeneity of the genetic material in the blood sample [52]. The low concentration of parasites in the samples from patients in the chronic phase makes it necessary to homogenize the genetic material to ensure that after collecting the volume for the extraction process, the highest possible concentration of free parasite DNA is achieved. The search for new methods capable of increasing the ability to dissociate and disperse DNA throughout the sample seems to be the way forward in clinical practice.

No significant differences were found in parameters such as age, location, endemicity, type of study, reference serological tests, or year of publication. The volume of sample processed for DNA extraction did not turn out to be a factor involved in the heterogeneity of the studies either, in the same way as the different extraction techniques (salting out, phenol‒chloroform method or commercial methods), the different sample conservation protocols, or the bath in boiling water.

It should be noted that no significant differences were observed between the different main molecular techniques (cPCR, qPCR and LAMP) or in their variants, such as nested PCR, hot-start techniques, multiplex PCR or duplex PCR. The different molecular targets do not appear to be determinant in the diagnosis of the disease either, although this result is not entirely clear, as there exist a vast majority of articles in which kDNA and satDNA detection techniques are assessed. We can conclude that both targets have similar diagnostic efficacy, but in the case of the other molecular targets such as H2DNAA, Tc24, pE13DNA or 18SrRNA, no conclusion could be drawn due to the scarcity of articles that analysed techniques focused on these genomic regions.

A large number of different primer sets have been defined in the different bibliographic records, but it was not possible to identify the different combinations as a source of heterogeneity. Furthermore, the differences between the articles that used different types of development of the results or development using fluorescence or turbidimetry techniques in real time were not significant.

### Limitations of evidence

There is a specific probability that the observed heterogeneity comes from items not included in this analysis due to incomplete data in the included publications. In many studies, methodological characteristics, such as sample collection period, age or mean age of participants, sex distribution of the sample, rural or urban origin, clinical presentation of the disease, sample preservation conditions, period between compared tests, DTUs endemic to the different geographic areas at the time of the study, inhibition and reaction contamination controls, or the time between sample collection and processing, were not determined/specified/recorded. In addition, summary estimates of diagnostic efficacy were grouped into subgroups under conditions of high heterogeneity; therefore, we should be careful/cautious/conservative in the interpretation of the results.

### Applicability of findings/Interpretation

We recommend that health authorities standardize and optimize molecular diagnostic techniques for better diagnosis and control of this disease.

## Conclusions

The results of this systematic review and meta-analysis indicate the following: 1st) In chronic patients, the performance of molecular techniques is not good enough for diagnosis. In patients in the acute phase, reliability increases significantly. These results support the conclusion that molecular techniques can be applied for routine diagnosis in patients in the acute phase, but the simultaneous use of molecular and serological techniques in chronic patients is recommended. 2nd) Among the different molecular techniques analysed, a high variability of diagnostic efficiency is observed. Parasitemia is one of the most important limiting factors since it influences diagnostic efficacy, and it is also a highly variable condition in each patient. Likewise, the different protocols applied in molecular tests involve a large number of factors that could increase the differences in the results. 3rd) The results described in this meta-analysis (qualitative and quantitative analyses) do not allow the selection of the optimal protocol of molecular method for the study of *Trypanosoma cruzi* infection in any of its phases, among other reasons due to the complexity of this infection.

### Supplementary Information

**Additional file 1: Table S1.** PCR: characteristics of studies included in the systematic review.

**Additional file 2: Table S2.** LAMP: characteristics of studies included in the systematic review.

**Additional file 3: Figure S1.** PRISMA flowchart of the literature study process and selection.

## Data Availability

All data generated or analysed during this study are included in this published article. The data presented in this study are available upon reasonable request from the corresponding author.

## Abbreviations

ACD: Acute phase Chagas Disease
CD: Chagas Disease
CMIA: Chemiluminescent microparticle immunoassay test
CCC: Chronic Chagasic cardiomyopathy
CCD: Chronic phase Chagas disease
DOR: Diagnostic odds ratio
DTUs: Discrete typing units
EDTA: Ethylenediaminetetraacetic acid
ELISA: Enzyme-linked immuno sorbent assay
GEB: Guanidine-EDTA-blood
HSROC: Hierarchical summary receiver operating characteristic
ICT test: Immunochromatography test
kDNA: Kinetoplast DNA
LR: Likelihood ratio
LAMP: Loop-mediated isothermal amplification
NTD: Neglected tropical disease
PAHO: Pan American Health Organization
PRISMA: Preferred Reporting Items for Systematic reviews and Meta-Analyses
PCR: Polymerase chain reaction
QUADAS-2: Quality Assessment of Diagnostic Accuracy Studies tool
qPCR: Quantitative PCR
satDNA: Satellite DNA
WHO: World Health Organization
