# Supplementary material for: Molecular diagnosis of Chagas disease: a systematic review and meta-analysis
Source: Infect Dis Poverty. 2023 Oct 16;12:95. doi: 10.1186/s40249-023-01143-7 (PMC10577976; doi:10.1186/s40249-023-01143-7)
Supplement: Supplementary file 1 — Additional file 1: Table S1. PCR: characteristics of studies included in the systematic review. [file 40249_2023_1143_MOESM1_ESM.docx]

**Additional file 1:**

**Table S1. PCR:** characteristics of studies included in the systematic review.

| Study ID | | Population | | | | | | Technique description | | | | | | | | Outcomes | |
| --- | --- | --- | --- | --- | --- | --- | --- | --- | --- | --- | --- | --- | --- | --- | --- | --- | --- |
| First author, year [Ref.] | **Design study** | **Country** | **Period data collection** | P**articipants** | **Sample size** | **Infected** | **Un-infected** | **Sample Volume** | **Guanidine** | **Boiling water bath.** | **Extraction** | **Molecular Technique** | **Target Sequence** | **Primer (Probe)** | **Master Mix** | **Sensitivity value** | **Specificity value** |
| Benatar, 2021 [18] | Prospective multicenter study; follow-up at birth, 8 weeks, and 9 months | Argentina | No data | Infants born to CD mothers at endemic and non-endemic sites of Argentina | 370 complete followed-up; but  246 samples with qPCR accuracy | ACD: 13 | 235 | 1-5 | Yes | Yes | Fiberglass Columns (Kit) | qPCR | satDNA | No data | Wiener  (Kit) | 76.2% (50.17-100.00) | 92.83% (89.44-96.22) |
| Bisio, 2021 [20] | A prospective cohort study | Argentina | 2012-2015 | Infants under 9 months of age | 120 were enrolled, 102 of them fulfilled the follow-up | ACD: 13 | 89 | 0.5–2 | Yes | Yes | Fiberglass Columns (Kit) | qPCR | satDNA | Cruzi 1, Cruzi2 and (Cruzi 3) | Roche (Kit) | 100.0% (75.3–100.0) | 100.0% (95.9–100.0) |
| Britto, 1995 [21] | Recruiting the last 15 years | Brazil | No data | Individuals from northeastern Brazil | 172 | CCD: 47 | 125 | 5 | Yes | Yes | Phenol-Chloroform | cPCR | kDNA | 121 and 122 | Roche (Kit) | 52,5% | 100% |
| Carriazo, 1998 [5] | No data | Argentina | No data | CCD patients | 26 | CCD: 16 | 10 | No data | Yes | Yes | Phenol-Chloroform | cPCR | pE13DNA | O1 and O2 | No data | 100% | 100% |
| Castro, 2002 [22] | Patients and control group | Brazil | No data | Untreated CCD patients | 69 | CCD: 60 | 9 | 10 | Yes | Yes | Phenol-Chloroform | cPCR | kDNA | 121 and 122 | Sigma, USA | 86.7% | 100% |
| Cura, 2017 [23] | A retrospective comparative study (Case-control) | Argentina | 2000-2004 | Adults and infants | 47 infants  51 adults  98 total | 17 infants (ACD)  26 adults  43 total | 30 infants  25 adults  55 total | 0.5-5 | Yes | Yes | Fiberglass Columns | cPCR  qPCR | kDNA  satDNA | 121 and 122  23F, 148R and (71P)  Tcz 1 and Tcz2 Cruzi 1 Cruzi2 and (Cruzi 3) | Roche (Kit) | SatDNA: qPCR: 60% (46-74); cPCR: 40% (26-54).  kDNA: 67% (53-80) cPCR: 58% (43-72) | SatDNA: qPCR: 96% (88-99); cPCR: 100% (93-100)  kDNA: qPCR: 80% (68-88) cPCR: 93% (83-97) |
| De Winne, 2014 [24] | A prospective study | Chile, Argentina and Spain | Chile, 2004, 2009 and 2011/  Argentina, 2008-2010/  Spain, 2011 | CCD patients >12 years of age and Healthy endemic controls | 275 | CCD: 187 | 88 | 2.5-5 | Yes | No | Fiberglass Columns | qPCR | kDNA  satDNA | No data | OligoC-T Coris (Kit) | kDNA OligoC-TesT: 79.1% (72.8%–84.4%)  satDNA OligoC-TesT: (67.9%, 60.9%–74.2%) | satDNA OligoC-TesT: 99.1% (95.2%–99.8%)  kDNA OligoC-TesT 97.4% (92.6%–99.1%) |
| Deborggraeve, 2009 [25] | A longitudinal study | Chile | 2000-2004 | Adults and children | 141 | CCD: 27 (infected adults)  ACD: 6 (infected children) | 108 (60 Chagas non-endemic and 48 endemic control persons) | 2 | Yes | Yes | Silica gel columns | qPCR | satDNA | No data | OligoC-T Coris (Kit) | ACD:66.7% (30%–90.3%)  CCD:100% (87.5%–100%) | 100% (92.6%–100%) |
| Diez, 2008 [6] | A prospective study | Argentina | No data | Total samples 121/  A single sample at birth104/  With follow-up 3 samples (2-4-9 months) 17  newborns from mothers with positive T.cruzy serology | 17 | ACD: 5 | 12 | 2 | Yes | Yes | Phenol-Chloroform | cPCR | kDNA | 122 and 122 or 256 | Chagatest; Wiener Laborato-ries, Rosario, Argentina (IHA kit);  Wiener Laboratory (ELISA kit) | 100% | 100% |
| Duarte, 2014 [26] | A case-control design | Colombia | No data | Patients with Chagasic cardiomyopathy | 205 | CCD: 100  patients with Chagasic cardio-myopathy | 105 | 4 | No | No | N.D. | cPCR | kDNA  satDNA | 121 and 122  Tcz 1 and Tcz2 | No data | PCR kDNA: 51% (41-61)  PCR nDNA: 22% (14-31) | PCR kDNA: 100% (97-100)  PCR nDNA: 100% (97-100) |
| Espinoza, 1996 [27] | No data | Bolivia | No data | Bolivian children living in endemic area | 22 | CCD: 17  Bolivian children living in endemic area | 5 | No data | Yes | Yes | Phenol-Chloroform | cPCR | kDNA  Tc24DNA | 121 and 122 | APPLIGENE, Illkirch, France | Tc24:100  kDNA:94.1% | 100% |
| Ferrer, 2013 [28] | Case-control | Venezuela | No data | Sample of ACD/CCD patients from Venezuela | 111 | CCD: 42  ACD: 39 | 30 | No data | No data | Yes | Chelex Resin | cPCR | kDNA  satDNA | 121 and 122  Tcz 1 and Tcz2 | Sigma, St. Louis, MO, EE.UU;  Promega, Madison, EE.UU | ACD:  kDNA:76,9  satDNA:79,5  CCD:  kDNA:23,8  satDNA 26,2 | 100% |
| Gil, 2007 [29] | Concordance study | Colombia | No data | No data | 156 | CCD: 89 | 67 | 5 | No | No | Phenol-Chloroform | cPCR | H2ADNA | TcH2AF and TcH2AR | GFX™ Genomic Blood DNAPurification Kit | 88% (75%-95%) | 92,5% (87.7%-97.2%) |
| Gomes, 1999 [7] | No data | Brazil | No data | Individuals who had lived in CD endemic areas | 113 | CCD: 79 | 34 | 15 | Yes | Yes | Fiberglass Columns | cPCR | kDNA | 121 and 122 | Sigma Chemical Company, St. Louis, MO | 83,5% | 70% |
| Gutierrez, 2004 [30] | No data | Colombia | No data | No data | 120 | CCD: 79 confirmed seropositive cases | 25 validated seronegative individuals and 16 individuals with discordant serological results | 5 | Yes | Yes | Phenol-Chloroform | cPCR | kDNA | 121 and 122 | - | 84,8% | 96% |
| Hernández, 2016 [31] | The sample collection was both retrospective (for the period 2004–2011) and prospective (for the period 2012–2015) | Colombia | 2004-2015 | No data | 708 | CCD: 481  ACD: 71 | 156  (141 chronic phase/ 15 febrile negatives in acute phase) | 10 | Yes | No | Fiberglass Columns | cPCR  qPCR | satDNA | Tcz 1 and Tcz2  Cruzi 1, Cruzi2 and (Cruzi 3) | High Pure PCR Template Roche kit | ACD:  qPCR 95.7 (88.3–98.5)  cPCR 84.5 (74.3–91.2)  CCD:  qPCR: 64.2 (59.8–68.4)  cPCR 56.8 (52.3–61.1) | ACD:  qPCR:100.0 (79.6–100.0)  cPCR:100.0 (79.6–100.0)  CCD:  qPCR:97.1 (92.9–98.8)  cPCR:97.9 (93.9–99.2) |
| Mayta, 2019 [32] | Archived samples | Bolivia | No data | Archived samples from women presenting for delivery | 265 | CCD: 150 | 115 | No data | Yes | No | Fiberglass Columns (Kit) | qPCR | satDNA | Cruzi 1, Cruzi2 and Cruzi 3 | Roche (Kit) | 31.3% (24.0–39.4%) | 100% (96.8–100%) |
| Melo, 2015 [33] | A case-control study | Brazil | 2004-2015 | No data | 60 | CCD: 40 | 20 individuals without T.*cruzi* infection living in non-endemic CD areas | 5 | No | No | QIAamp Yeslice Gel Columns (Kit) | qPCR | satDNA | Cruzi 1, Cruzi2 and Cruzi 3 | Applied Biosystem (Kit) | 97,5% | 100% |
| Messenger, 2017 [34] | A cohort study of pregnant women and their infants in 2 hospitals using 3 techniques | Bolivia | 2010-2014 | No data | 282  (487 infants of 476 seropositive women) | ACD: 25  (38 infants of 35 mothers (32 infections were detected by qPCR in the first month of life) | 257 | No data | Yes | No | Phenol-Chloroform | qPCR | satDNA | Cruzi 1, Cruzi2 and Cruzi 3 | In maternal infection: Wiener Laboratories, Rosario, Argentina.  In congenital infection: Qiagen, Hilden, Germany | 76% (55-91) | 100% (99-100) |
| Mora, 2005 [35] | Each subject was then followed serologically | Argentina | No data | Umbilical cord blood from newborns (n = 302) to infected mothers was analyzed with microhematocrit, hemoculture, and PCR methods | 169 | ACD: 23 | 146 | 2–5 | Yes | Yes | Phenol-Chloroform | cPCR | kDNA | 121 and 122 | In-House | 34,8% | 97,3% |
| Piron, 2007 [36] | No data | Spain | 2000-2004 | Adults | 183 | CCD: 38 adults (native to endemic areas (33 Bolivians, 3 Argentines, 1 Brazilian, 1 Honduran) and living in Barcelona)  ACD: 1 a child with an acute congenital infection | 144  (100 healthy individuals from endemic areas, 24 healthy individuals from non-endemic areas and 20 patients with Leishmaniasis) | 1 | Yes | No | Fiberglass Columns | Nested PCR | satDNA | TcZ1, TcZ2, TcZ3 and TcZ4 | High Pure PCR Template Preparation kit (Roche, Basel, Switzerland) | N-PCR 42%  qPCR 42% | 100% |
| Ramírez, 2009 [37] | A case-control study | Colombia | 1997-2001 | Adult patients in the chronic phase | 260 | CCD: 240 | 20 | 10 | Yes | Yes | Phenol-Chloroform | cPCR | kDNA  satDNA | 121 and 122 | commercial kit Chagatest, Wiener Lab, Rosario, Argentina | kDNA: 70%  satDNA: 75% | 100% |
| Ramírez, 2015 [11] | International study from 14 countries | Mexico, French Guiana, Bolivia, Venezuela, Colombia, Brazil, Argentina, and Spain | 2015-09 | Adult and minor subjects | 206 | 156  CCD: 145  ACD: 11 | 50  persons from Argentina with negative serology for T.cruzi | No data | Yes | Yes-No | Fiberglass Columns | MTqPCR | kDNA  satDNA | 23F, 148R and 71P  Cruzi 1, Cruzi2 and Cruzi 3 | Roche (Kit) | CCD:  SatDNA: 80.69%;  kDNA: 84.14%,  ACD:  SatDNA and kDNA qPCR 100% | 100% |
| Schijman, 2003 [38] | A prospective study in congenital CD born to seroreactive mothers living in a non-endemic area | Argentina | 1998-2000 | Children born to infected mothers | 124  152  GA: 50 infants aged 0–6 months/ GB: 102 children aged 7 months to 17 years | ACD: 61  78  GA: 17  GB: 61 | 63  74  GA: 33  GB:41  GC: 22 infants born to non-infected mothers | 2 | Yes | Yes | Membrana DNeasy Quagen (Kit) | cPCR | kDNA | 121 and 122 | Sigma, St Louis, USA;  Promega, WI, USA | 73,8% | 100% |
| Seiringer, 2017 [39] | Longitudinal compared four distinct PCR methods for detection of T.cruzi | Spain | 2010-2013 | Patients in chronic phase | 53 | CCD: 50 | 3 | No data | Yes | Yes | Fiberglass Columns | cPCR  qPCR  (Com) qPCR | kDNA  satDNA | TcZ1 and Tcz2 | High Pure PCR Template Preparation kit (Roche Diagnostics Corp., Indianapolis, IN, USA | cPCR:  Method A: 100%;  Method B: 94%  qPCR:  Method C: 90%;  Method D: 98% | 100% |
| Simón, 2019 [40] | An observational longitudinal study in congenital CD | Spain | January 2007-December, 2017 | Children born in Spain from Latin American Chagas-infected mothers | ACD: 188 | ACD: 11 | 177 | 2 | Yes | Yes | Maxwell 16 Blood DNA Purification (Kit) | cPCR | kDNA | 121 and 122 | Maxwell 16 Blood DNA purification Kit (Promega Biotech Iberica) | 90.9 (62.3–98.4) | 100 (97.9–100) |
| Velázquez, 2014 [41] | Longitudinal prospective | Argentina | 2004-2009 | Children | 468  ACD: 337 (123+8 were excluded did not complete the follow-up) | ACD:41 | 269 | 2 | Yes | Yes | Phenol-Chloroform | cPCR | kDNA | 121 and 122 | No data | 100% (IC: 89,33-99,78) | 100% (IC: 98,40-99,97) |
| Viettri, 2022 [42] | Samples evaluated | Venezuela | No data | Blood and serum patients in the acute and chronic phase vs. patients with other diseases and healthy individuals | 129 | ACD: 35  CCD: 33 | 61  (31 other diseases and 30 healthy) | No data | No | Yes | Chelex Resin | cPCR  qPCR | satDNA | Tcz 1 and Tcz 2.  No data | Vircell®, Granada, Spain | cPCR: 91.2 % (82.1–95.9)  Commercial qPCR: 92.6 % (83.9–96.8) | In House cPCR: 100 % (94.1–100)  Commercial qPCR: 100 % (94.1–100) |
| Wehrendt, 2021 [43] | Longitudinal prospective; Follow-up at birth, 2 and 9 months | Bolivia | No data | Blood samples from neonates born to seropositive mothers | 25 | ACD: 10 congenitally infected | 15 | No data | Yes | Yes | Fiberglass Columns (Kit) | qPCR | satDNA | Cruzi 1, Cruzi2 and Cruzi 3 | Roche (Kit) | 80% | 100% |
