# Supplementary material for: Molecular diagnosis of Chagas disease: a systematic review and meta-analysis
Source: Infect Dis Poverty. 2023 Oct 16;12:95. doi: 10.1186/s40249-023-01143-7 (PMC10577976; doi:10.1186/s40249-023-01143-7)
Supplement: Supplementary file 2 — Additional file 2: Table S2. LAMP: characteristics of studies included in the systematic review. [file 40249_2023_1143_MOESM2_ESM.docx]

**Additional file 2:**

**Table S2. LAMP:** characteristics of studies included in the systematic review.

| Study ID | | Population | | | | | | Technique description | | | | | | | | Outcomes | |
| --- | --- | --- | --- | --- | --- | --- | --- | --- | --- | --- | --- | --- | --- | --- | --- | --- | --- |
| Author, year [Ref.] | **Study design** | **Country** | **Period of data collection** | **Participants** | **Sample size** | **Infected** | **Uninfected** | **Sample Volume** | **Guanidine** | **Boiling water bath.** | **Extraction** | **Molecular Technique** | **Target Sequence** | **Primer (Probe)** | **Master Mix** | **Sensitivity value** | **Specificity value** |
| Besuschio, 2017 [9] | Case-Control | Argentina | 2003-2016 | Neonates (5ACD),  transplant. (3ACD)  10CCD,  Immunosuppression after organ transplanted (5CCD) | 33 | ACD: 8  CCD: 15 | 10 | No data | No data | Yes | Fiberglass Columns | LAMP | satDNA | No data | qPCR: Roche (Kit);  LAMP: Eiken | CCD:60%  ACD:100% | CCD:100%  ACD:100% |
| Besuschio, 2020 [19] | Retrospective study | Argentina, Venezuela | 2013-2017 | Infants (ACD) born to seropositive mothers | 30 | ACD: 13 | 17 | 1.5 | Yes | Yes | Fiberglass Columns | LAMP | satDNA | No data | qPCR: Roche (Kit);  LAMP: Eiken | 100% | 100% |
| Bisio, 2021 [20] | Prospective cohort study | Argentina | 2012-2015 | Infants under 9 months of age | 102 of 120 fulfilled the follow-up | ACD: 13 | 89 | 0.5-2 | Yes | Yes | Fiberglass Columns | LAMP | 18S rRNA | FIP and BIP, LF and  LB, F3 and B3. | qPCR: Roche (Kit) | 69.2% (38.6–90.9) | 100.0% (95.9–100.0) |
| Flores-Chavez, 2021 [8] | Retrospective study (case-control) | Spain | No data | Congenital (ACD) and CCD | 295 | ACD: 39  CCD: 174 | 82 | No data | Yes | Yes | Fiberglass Columns | LAMP | satDNA | No data | qPCR: Roche (Kit) | CCD:47%  ACD:97% | CCD:100%  ACD:94% |
| Wehrendt, 2021 [43] | Longitudinal prospective; Follow-up at birth, 2 and 9 months | Bolivia | No data | Blood samples from neonates born to seropositive mothers | 25 | ACD: 10 congenitally infected | 15 | No data | Yes | Yes | Magnetic particles | LAMP | satDNA | No data | qPCR: Roche (Kit) | 100% | 100% |
