# Supplementary material for: Molecular diagnosis of Chagas disease: a systematic review and meta-analysis
Source: Infect Dis Poverty. 2023 Oct 16;12:95. doi: 10.1186/s40249-023-01143-7 (PMC10577976; doi:10.1186/s40249-023-01143-7)
Supplement: Supplementary file 3 — Additional file 3: Figure S1. PRISMA flowchart of the literature study process and selection. [file 40249_2023_1143_MOESM3_ESM.docx]

**Identification of studies via databases and registers**

**Identification of studies via other methods**

Records identified through database searching (**n = 858**):

PubMed (n = 363)

Web of Science (n = 187)

Embase (n= 175)

Scopus (n= 117)

Lilacs (n= 16)

Records identified from:

Citation searching (**n = 30**)

Records removed before screening (**n = 611**):

Duplicate records removed (n = 533)

Records marked as ineligible by automation tools (n = 35)

Records removed for other reasons (n = 43)

**Identification**

Records excluded

(**n = 21**)

Records screened

(**n = 247**)

Records excluded

(**n = 52**)

Reports not retrieved

(**n = 6**)

Reports not retrieved

(**n = 19**)

Reports sought for retrieval

(**n = 195**)

**Screening**

Reports excluded (**n = 147**):

Treatment Monitoring (n = 13)

Immunocompromised patients (n = 9)

Lack of diagnostic accuracy data (n= 69)

Sample different from blood sample (n= 56)

Reports assessed for eligibility

(**n = 176**)

Reports assessed for eligibility

(**n = 3**)

Studies included in review

(**n = 29**)

Total studies included in review

(**n = 32**)

**Included**

**Additional file 3:**

**Figure S1.** PRISMA flowchart of the literature study process and selection
